# Insights into Phosphate Cooperativity and Influence of Substrate Modifications on Binding and Catalysis of Hexameric Purine Nucleoside Phosphorylases

**DOI:** 10.1371/journal.pone.0044282

**Published:** 2012-09-05

**Authors:** Priscila O. de Giuseppe, Nadia H. Martins, Andreia N. Meza, Camila R. dos Santos, Humberto D’Muniz Pereira, Mario T. Murakami

**Affiliations:** 1 Laboratório Nacional de Biociências (LNBio), Centro Nacional de Pesquisa em Energia e Materiais, Campinas, São Paulo, Brazil; 2 Instituto de Física de São Carlos, Grupo de Cristalografia, Universidade de São Paulo, São Carlos, São Paulo, Brazil; Griffith University, Australia

## Abstract

The hexameric purine nucleoside phosphorylase from *Bacillus subtilis* (BsPNP233) displays great potential to produce nucleoside analogues in industry and can be exploited in the development of new anti-tumor gene therapies. In order to provide structural basis for enzyme and substrates rational optimization, aiming at those applications, the present work shows a thorough and detailed structural description of the binding mode of substrates and nucleoside analogues to the active site of the hexameric BsPNP233. Here we report the crystal structure of BsPNP233 in the apo form and in complex with 11 ligands, including clinically relevant compounds. The crystal structure of six ligands (adenine, 2′deoxyguanosine, aciclovir, ganciclovir, 8-bromoguanosine, 6-chloroguanosine) in complex with a hexameric PNP are presented for the first time. Our data showed that free bases adopt alternative conformations in the BsPNP233 active site and indicated that binding of the co-substrate (2′deoxy)ribose 1-phosphate might contribute for stabilizing the bases in a favorable orientation for catalysis. The BsPNP233-adenosine complex revealed that a hydrogen bond between the 5′ hydroxyl group of adenosine and Arg^43*^ side chain contributes for the ribosyl radical to adopt an unusual C3’-*endo* conformation. The structures with 6-chloroguanosine and 8-bromoguanosine pointed out that the Cl^6^ and Br^8^ substrate modifications seem to be detrimental for catalysis and can be explored in the design of inhibitors for hexameric PNPs from pathogens. Our data also corroborated the competitive inhibition mechanism of hexameric PNPs by tubercidin and suggested that the acyclic nucleoside ganciclovir is a better inhibitor for hexameric PNPs than aciclovir. Furthermore, comparative structural analyses indicated that the replacement of Ser^90^ by a threonine in the *B. cereus* hexameric adenosine phosphorylase (Thr^91^) is responsible for the lack of negative cooperativity of phosphate binding in this enzyme.

## Introduction

Purine nucleoside phosphorylases (PNPs; EC 2.4.2.1) are versatile enzymes that catalyze the reversible phosphorolysis of purine (2′deoxy)ribonucleosides producing bases and (2′deoxy)ribose 1-phosphate [1]. Their key role in the purine salvage pathway made PNPs attractive targets for drug design against several pathogens, such as *Mycobacterium tuberculosis* [2], [3], *Plasmodium falciparum* [4]–[7], *Trichomonas vaginalis* [8]–[10] *and Schistosoma mansoni* [11], [12], which lacks the *de novo* pathway for purine nucleotides synthesis. Due to their catalytic function, PNPs have also been investigated for the synthesis of nucleoside analogues (NAs) [13] and the activation of prodrugs in anti-cancer gene therapies [14].

NAs can be used in the treatment of a range of human viral infections, such as those caused by HIV, herpesvirus and hepatitis B/C virus [15]–[19]. They are among the first cytotoxic molecules to be used in the treatment of cancer [20] and have been studied as potential drugs against tuberculosis [21], [22], malaria [7], [23], trichomoniasis [24] and schistosomiasis [25]. The chemical synthesis of these compounds is generally a costly multistep process that includes several protection and deprotection stages [13], [26]. This has encouraged the development of new methods for the synthesis of NAs using PNPs and other enzymes as biocatalysts [13], [27], [28]. The main advantages of this approach are the higher stereospecificity, regioselectivity and efficiency of enzymes, whose employment usually dispenses group protection and purification steps, optimizing the process [13].

The differences in substrate specificity regarding trimeric and hexameric PNPs have allowed the development of suicide gene therapies strategies against solid tumors [14], [29]. Trimeric PNPs are mainly found in mammalian species and are specific for guanine and hypoxanthine (2′-deoxy)ribonucleosides whereas hexameric PNPs are prevalent in bacteria and accept adenine as well as guanine and hypoxanthine (2′-deoxy)ribonucleosides as substrates [1]. Thus, nontoxic adenosine analogues, which are poor substrates for human PNP, can be cleaved to cytotoxic bases specifically in tumor cells transfected with the bacterial hexameric PNP gene [14]. Main advances in this field have been achieved with the *E. coli* PNP [30]–[33].

In this context, the aim of the present work was to shed light on how a diverse set of substrate modifications affects its binding and catalysis by hexameric PNPs using a structural approach. For this purpose, we choose the hexameric PNP (BsPNP233) from the model specie *Bacillus subtilis*, which displays great biotechnological potential to produce NAs, including the antiviral drug ribavirin [34]. We have solved the crystal structure of BsPNP233 in the apo form and in complex with 11 ligands comprising sulfate, bases, natural nucleosides and NAs, including clinically relevant compounds. The crystal structure of six ligands (adenine, 2′deoxyguanosine, aciclovir, ganciclovir, 8-bromoguanosine, 6-chloroguanosine) in complex with a hexameric PNP are presented for the first time.

Besides providing a broad structural basis for studies aiming at the rational design of BsPNP233 and its homologues for biotechnological applications, this work also bring new insights into the distinct kinetic models for phosphate binding in hexameric PNPs. Furthermore, the structural information showed here may also be instrumental for the development of new inhibitors against hexameric PNPs from pathogens such as *Plasmodium falciparum* and *Trichomonas vaginalis* [5], [6], [8], [9] and for the combined design of both hexameric PNPs and prodrugs to improve specificity and efficiency of anti-cancer PNP gene therapies [14].

## Materials and Methods

### Chemicals

Adenine (Ade), adenosine (Ado), 2-fluoradenosine (F-Ado), tubercidin (TBN), 2′-deoxyguanosine (dGuo), hypoxanthine (Hyp), ganciclovir (GCV), aciclovir (ACV), 8-bromoguanosine (Br-Guo) and 6-chloroguanosine (Cl-Guo) were all purchased from Sigma-Aldrich.

### Expression and Purification of Recombinant BsPNP233

BsPNP233 was expressed in *E. coli* cells and purified by immobilized metal affinity and size-exclusion chromatographies as described in [35]. The protein concentration was determined by absorption spectroscopy at 280 nm using the theoretical molar extinction coefficient of 16 515 M^−1^cm^−1^ calculated by the program ProtParam [36].

### Crystallization

BsPNP233 at 11 mg/ml in 20 mM Tris–HCl pH 7.0, 50 mM NaCl and 1 mM DTT was crystallized by sitting-drop vapor-diffusion technique according to conditions previously described [35]. The crystals belong to the space groups *P*32_1_, *P*6_3_22, *P*2_1_2_1_2_1_ and *H*32 with one, two or six monomers per asymmetric unit depending on symmetry and cell dimensions.

### Preparation of BsPNP233-ligand Complexes

The protein-ligand complexes were prepared by adding 0.1 µl of 50 mM ligand, dissolved in DMSO, to 1 µl crystallization drops at least 12 h prior to data collection. The ligands used were nucleosides, purine bases and NAs (Table S1). This procedure was performed in drops containing BsPNP233 crystals grown in 0.1 M sodium acetate pH 4.6, 3.2 M sodium chloride, 5% *(v/v)* glycerol at 291 K.

### X-ray Data Collection and Processing

X-ray diffraction experiments were performed on the W01B-MX2 beamline at the Brazilian Synchrotron Light Laboratory (Campinas, Brazil). The data collection was carried out using crystals soaked in a cryoprotectant solution composed by the mother liquor and 20% *(v/v)* glycerol and flash-cooled in a nitrogen-gas stream at 100 K. The radiation wavelength was set to 1.458 Å and a MAR Mosaic 225 mm CCD detector was used to record the X-ray diffraction data. Data were indexed, integrated and scaled using the HKL-2000 suite [37] or the programs MOSFLM [38] and SCALA [39] from the CCP4 package [40]. Data processing statistics are summarized in Table S1.

### Structure Determination and Refinement

The structures were solved by molecular replacement using the programs MOLREP [41] or PHASER [42], both from the CCP4 suite [40]. The first BsPNP233 structure was determined using the atomic coordinates of *B. anthracis* PNP (PDB code 1XE3) [43] as a search model. The subsequent BsPNP233 structures were solved using the atomic coordinates of BsPNP233 solved at 1.7 Å resolution (BsPNP233-GCV dataset, Table S1) as template. Refinement was carried out using the programs REFMAC5 [44] and COOT [45]. After 20 cycles of rigid body refinement in REFMAC5 [44], the models were refined alternating cycles of restrained isotropic refinement in REFMAC5 [44] and manual rebuilding and real space refinement in COOT [45]. Water molecules were added after refinement of the protein model at chemically reasonable places using COOT [45]. Subsequently, the ligands were added to the model and refined as described above using library descriptions generated by the program SKETCHER from the CCP4 suite [40]. The intensity based twin refinement of REFMAC5 was applied to refine the structures of BsPNP233 in complex with adenosine, 2-fluoradenosine and adenine. The majority of models for the BsPNP233 protein included all but the first and last residues (1 and 233). In the electron density map of the crystal structure solved in the space group *P*2_1_2_1_2_1_ the residue 1 and additional eight residues from the N-terminal his-tag were clearly defined and added to the model. Ramachandran analysis carried out by Molprobity [46] showed that all residues from all models are found in allowed regions (except Gly^121^ of the BsPNP233-Ade structure, chain B). Refinement statistics are detailed in Table S1. Weighted 2Fo-Fc maps (2mFo-DFcalc) of ligands as well as a table of interactions between ligands and protein residues are presented in the supplementary material (Figure S1, Table S2). The atomic coordinates and structure factors of form I (4D8V), form II (4D8X), form III (4D8Y), form IV (4D98) and the complexes of BsPNP233 with Hyp (4DAB), Ade (4DAO), Ado (4D9H), dGuo (4DA0), F-Ado (4DAN), Cl-Guo(4DAE), Br-Guo (4DA8), TBN (4DAR), GCV (4DA6) and ACV (4DA7) have been deposited in the Protein Data Bank, Research Collaboratory for Structural Bioinformatics, Rutgers University, New Brunswick, NJ (http://www.rcsb.org/).

### Figure Preparation

The figures of structures were prepared using PyMOL [47].

### Structural Alignment

All structural comparisons were performed using the SSM algorithm [48] available at the program COOT [45] or at the PDBeFold server [49].

## Results and Discussion

### BsPNP233 Conserves the Quaternary Structure and Topology of Hexameric PNPs

**Figure 1:**
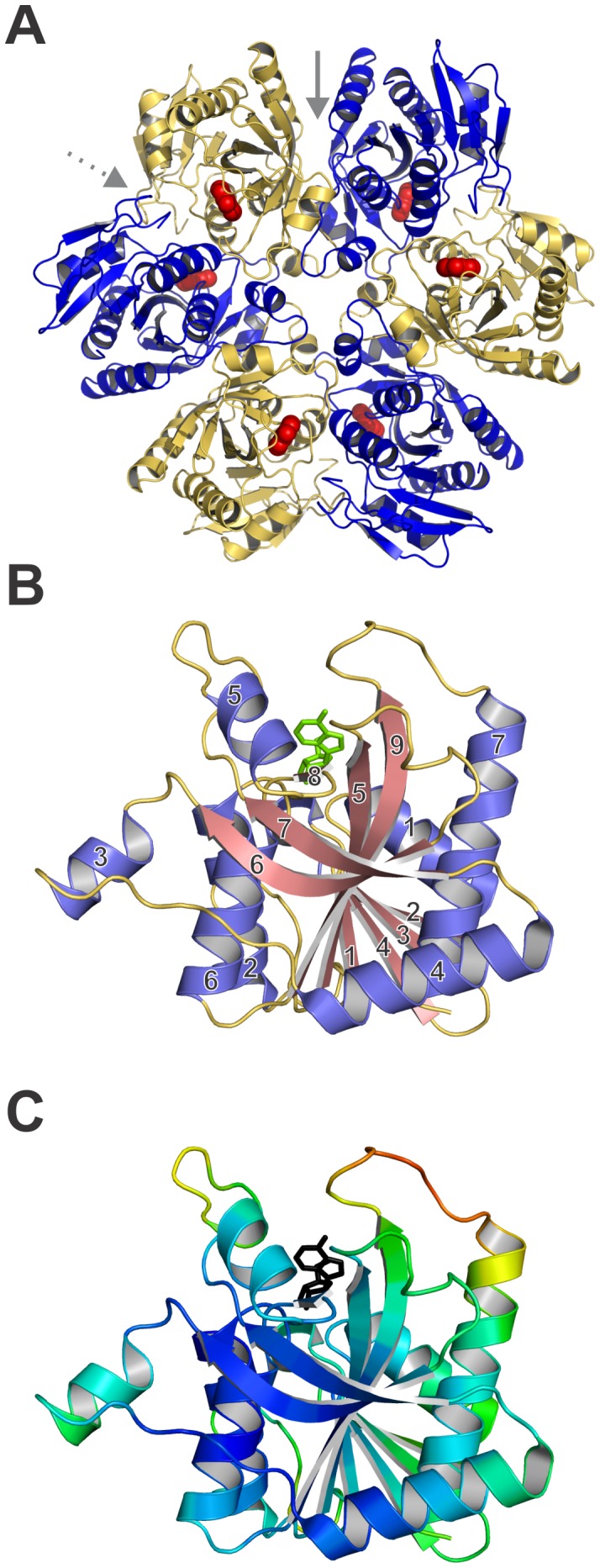
Overall structure of BsPNP233. A. Cartoon representation of the hexamer BsPNP233 with adenine (red spheres) bound in the active site. Solid and dashed grey arrows indicate the inter-dimeric and catalytic interfaces, respectively. B. Cartoon representation of BsPNP233 protomer in complex with adenosine (*green* stick). Loops, α-helices and β-strands are shown in *yellow*, *blue* and *pink*. The α-helices and β-strands were numbered according to the Mao and colleagues notation [50]. C. BsPNP233 protomer colored by B-factors from *dark blue* (lowest) to *red* (highest). Adenosine is represented by a *black* stick.

The crystal structure of BsPNP233 confirmed that it is a homohexamer with D_3_ symmetry as observed for other hexameric PNPs (Figure 1A) [50], [51]. It was solved by X-ray crystallography in four distinct space groups (*P*32_1_, *P*2_1_2_1_2_1_, *P*6_3_22 and *H*32). The crystal contacts are similar in the crystal structures solved in *P*32_1_, *P*2_1_2_1_2_1_ and *P*6_3_22 but differ in the *H*32 space group. In the later, we observed additional crystallographic interfaces, resulting from a more compact crystal packing with a lower solvent content (41%) than crystals belonging to other space groups (∼56%) (Figure S2) [35].

The BsPNP233 subunits surround a central axis and alternate in an up/down fashion forming a disc-shaped structure with six active sites: three located at the top face and other three at the bottom face. Analogously to other hexameric PNPs, BsPNP233 is a trimer of dimers where each subunit interacts with the adjacent subunits forming two interfaces: the catalytic, which contains the active site, and the inter-dimeric, involved in hexamer stabilization (Figure 1A). The inter-dimeric interface is larger than the catalytic interface and both are mainly maintained by hydrophobic interactions. In the ligand-free crystal structure (form II), the inter-dimeric and the catalytic interface areas are 1711 and 1554 Å^2^, respectively.

The BsPNP233 subunit conserves the *E. coli* hexameric PNP (EcPNP) subunit topology with few exceptions. Its central mixed β-sheet lacks the short β10 strand observed in EcPNP [50] and is surrounded by eight (instead of seven) α-helices (Figure 1B). The extra 5-residues α-helix connects the strands β2 and β3 and is not labeled to preserves the Mao and colleagues notation [50]. BsPNP233 and EcPNP subunits share sequence identity of 58% (PDB code 1ECP, [50]) and superpose with a r.m.s.d of 0.93 Å for 231 Cα atoms aligned (Figure S3). Structural alignment of BsPNP233 subunit with hexameric PNPs subunits from other *Bacillus* species resulted in a r.m.s.d of 0.80 Å - 0.94 Å for 231 Cα atoms aligned and an average sequence identity of 71% (Figure S3).

Analysis of the B-factor distribution in the apo BsPNP233 crystal structure shows that the loop connecting β9 and α7 as well as the N-terminal portion of α7 present the highest B-factor values, highlighting its intrinsic flexibility. As this region surround the active site, its flexibility may be important for catalysis (Figure 1C).

### Free Purine Bases Adopt Alternative Conformations in the Active Site

**Figure 2:**
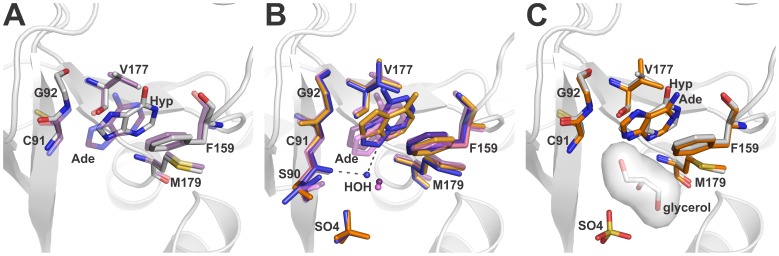
Comparison of free bases bound to the BsPNP233 active site. A. Structural comparison of a representative BsPNP233-Ade complex (*purple* carbon atoms) with the BsPNP233-Hyp complex (*grey* carbon atoms). B. The structure of the four BsPNP233-Ade complexes solved independently are superimposed. The sulfate-free Ade-complexes are colored in *purple* (chain A) and *pink* (chain B) whereas the two independent complexes solved with sulfate bound (dataset I) are colored in *orange* (chain A) and *blue* (chain B). C. The structure of the Ade-complex where Ade presents an alternative conformation (carbon atoms in *orange*) is superimposed in the structure of Hyp-complex (carbon atoms in *grey*). The surface of the glycerol molecule present at the Hyp-complex is shown to evidence the influence of this molecule in the position and orientation of Hyp in the active site. The hydrogen bonds are shown as *dashed lines.*

The crystal structures of BsPNP233 in complex with hypoxanthine (Hyp) and adenine (Ade) showed that the purine-binding site consists of residues Cys^91^, Gly^92^, Phe^159^, Val^177^ and Met^179^. Hydrophobic interactions are predominant in the stabilization of both ligands (Figure 2A).

The BsPNP233-Ade binary complex was solved with (BsPNP233-Ade-SO_4_) or without (BsPNP233-Ade) sulfate ion and represent the first of their kind to be reported for hexameric PNPs. Two subunits were observed in the asymmetric unit of both crystal structures and all of them exhibited clear density for the ligand in the active site (Figure S1).

Superposition of BsPNP233-Ade and BsPNP233-Ade-SO_4_ complexes showed a preferential orientation of Ade in the base-binding site, except in one case where it is rotated by 49° around an axis perpendicular to the base plane (Figure 2B). This alternative orientation is not followed by significant conformational changes in the active-site residues (Figure 2B); however, it alters the solvation of the active-site pocket. In the alternative orientation, a crystallographic water molecule in the ribose-binding site is absent. This solvent molecule mediates a hydrogen bond between the AdeN^9^ atom and the carbonyl group of Ser^90^ in the presence of sulfate ion (Figure 2B).

Interestingly, the Hyp adopts an orientation similar to the alternative conformation of Ade (Figure 2C). In this case, a glycerol molecule is located in the ribose-binding site and seems to induce the displacement of Hyp, avoiding a steric clash with the HypN^9^ atom. This observation, along with those described above, suggests that binding of the co-substrate ribose-1-phoshate might contribute for stabilizing the base in the favorable orientation for catalysis.

### The Hydrogen Bond between the 5′ Hydroxyl Group of Ado and Arg^43*^ Side Chain Contributes for a Ribosyl C3′-endo Conformation

**Figure 3:**
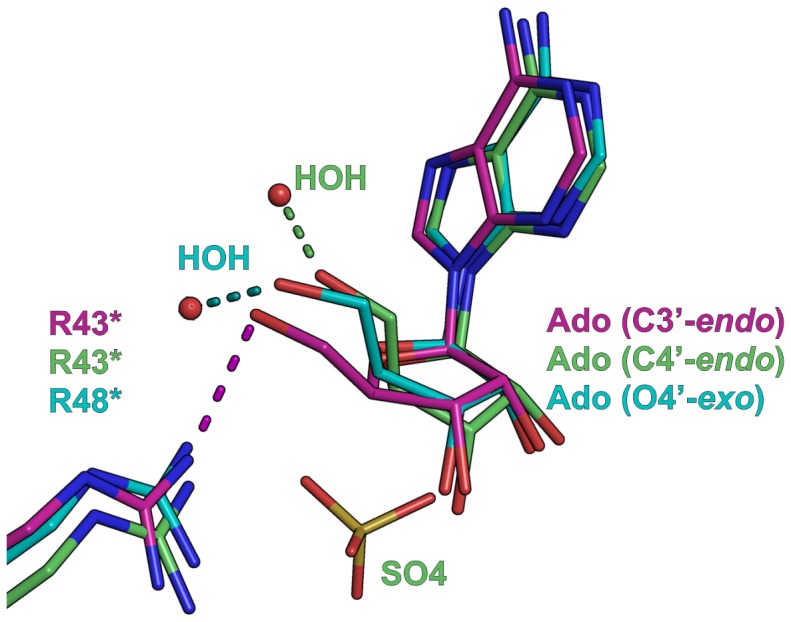
The different conformations of Ado ribosyl radical. Structural superposition of BsPNP233-Ado (*magenta* carbon atoms), *B. cereus* adenosine phosphorylase (BcAdoP)-Ado-SO_4_ (*green* carbon atoms, PDB code 3UAW [52]) and *Entamoeba histolytica* PNP-Ado (*cyan* carbon atoms, PDB code 3U40 [55]) complexes. The different puckers adopted by the ribose moiety of adenosine are labeled and the hydrogen bonds involving the 5′-OH group of Ado in each complex are represented by *dashed lines*. The sugar puckers were assigned by the pucker.py script of PyMOL [47].

The base moiety of adenosine (Ado) binds to the BsPNP233 active site in a very similar fashion to that seen in homologous PNPs (Figure 3). However, the ribosyl group adopts a C3′-*endo* form instead of the C4′-*endo* or O4′*-exo* conformations usually observed in Ado complexes with hexameric PNPs (PDB codes 3UAW, [52]; 1ODI, [53]; 1PK7, [54]; 3U40, [55]; 1Z37, [51]; 1VHW, [56]) (Figure 3). This unusual conformation may be explained by a hydrogen bond between the 5′ hydroxyl group of Ado and Arg^43*^ side chain (residues from the adjacent subunit are designated by an asterisk) not observed in other Ado complexes (Figure 3). Typically, the 5′-OH group of Ado is found interacting with one or two water molecules not observed in BsPNP233-Ado complex, suggesting that the hydration of the active site may influence the ribosyl conformation.

In sulfate/phosphate free complexes of hexameric PNPs with Ado, an O4′–*exo* conformation is usually found. However, in all complexes where the phosphate-binding site is occupied by a sulfate or phosphate, the ribosyl group shows a C4′–*endo* conformation, except for the *Thermus thermophilus* (TtPNP)-Ado complex (PDB code 1ODI, [53]). Since the side chain of Arg^43*^ participates in phosphate binding, the presence of this co-substrate and the hydration of the active site probably prevent the interaction between Ado 5′-OH group and Arg^43*^ side chain observed in BsPNP233-Ado complex favoring the ribose to adopt a C4′–*endo* conformation.

### 2′-deoxyguanosine Binding Mode Resembles to that Observed for Adenosine

**Figure 4:**
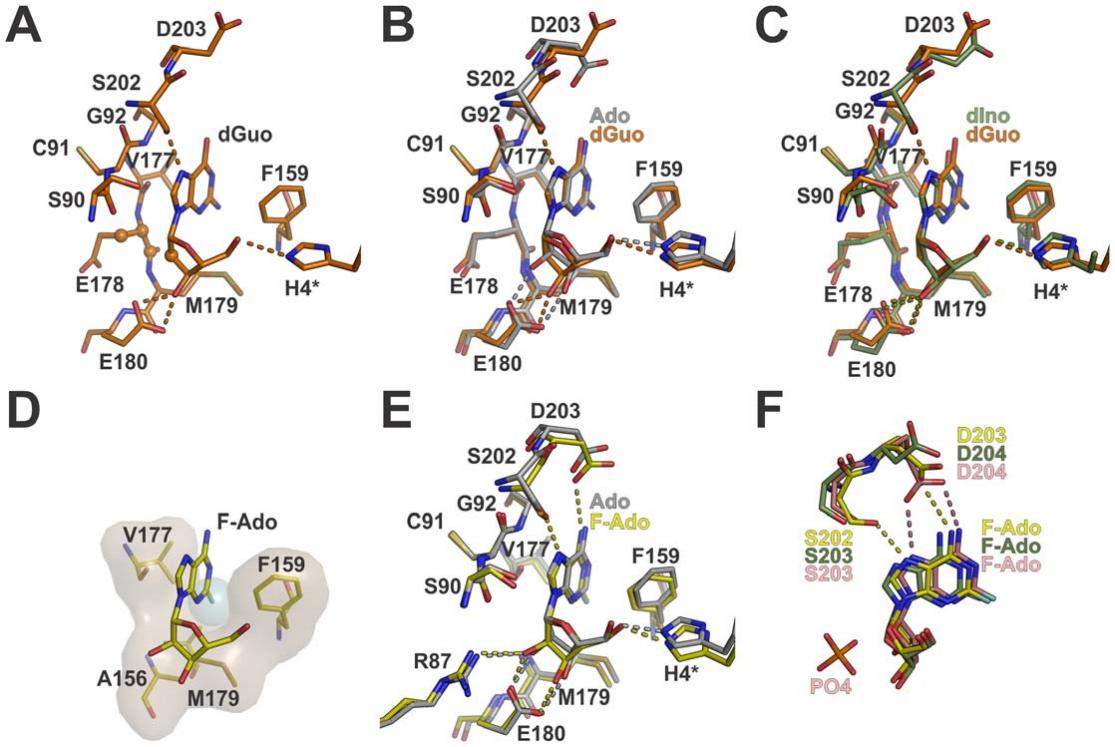
The binding mode of 2′ deoxyguanosine and 2-fluoradenosine. A. Representation of the BsPNP233 residues that interact with dGuo showing as spheres the atoms involved in hydrophobic interactions with dGuo C^2′^ atom. B. Structural alignment between the dGuo-complex (carbon atoms in *orange*) and the Ado-complex (carbon atoms in *grey*). C The structure of dGuo-complex and TvPNP-(2′-deoxyinosine) complex (carbon atoms in *green*, PDB code 1Z39, [51]) are superimposed. D. Representation of F-Ado complex showing the residues involved in van der Waals interactions with F^2^ atom (*light blue* sphere). E. Structural comparison of F-Ado complex (carbon atoms in *yellow*) with the Ado-complex (carbon atoms in *grey*). F. The structures of F-Ado complex, EcPNP-F-Ado-PO_4_ complex (carbon atoms in *pink*, PDB code 1PK9 [54]) and TvPNP-F-Ado complex (carbon atoms in *green*, PDB code 1Z35, [51]) are superimposed. In all panels hydrogen bonds are represented by *dashed lines* and color coded according to their respective structures.

In the BsPNP233-(2′-deoxyguanosine) complex structure, the base of 2′-deoxyguanosine (dGuo) binds to the active site in a similar manner to that observed for Ado (Figure 4). Neither the extra amino group at position 2 nor the carbonyl group at position 6 was observed making hydrogen bonds with the protein residues. A hydrogen bond between dGuoN^7^ and Ser^202^O^γ^ atoms slightly rotates the base and brings the residue Ser^202^ closer to the substrate (Figure 4B). The lack of the 2′-OH group in dGuo is counterbalanced by extra hydrophobic interactions between dGuoC^2′^ and Glu^178^ carbon atoms (Figure 4A). Comparisons between BsPNP233-dGuo and *T.* vaginalis PNP (TvPNP)-(2′-deoxyinosine) complexes showed that the deoxyribosyl group of both ligands conserves the binding mode, whereas the base assumes a little different orientation induced by the dGuoN^7^-Ser^202^O^γ^ hydrogen bond exclusively observed in BsPNP233-dGuo complex (Figure 4C).

### BsPNP233 can be Explored as an Alternative in Gene Therapy Approaches using 2-Fluoradenosine as Prodrug

The compound 2-fluoradenosine (F-Ado) is an adenosine analogue which liberates the toxic metabolite 2-fluoradenine when cleaved. Its deoxy form has been studied as a prodrug in an anti-tumor gene therapy approach based on a modified human PNP [57]. The crystal structure of BsPNP233-(F-Ado) complex had two BsPNP233 subunits per asymmetric unit and both presented clear electronic density for the ligand (Figure S1). In the two independent active sites, F-Ado was found in the same orientation, similar to that of Ado (Figure 4E). The extra fluorine atom at position 2 is allocated in a hydrophobic micro-environment consisting of Ala^156^, Phe^159^, Val^177^ and Met^179^ (Figure 4D). This motif is fully conserved in EcPNP, which has been tested in anti-tumor gene therapy by activating produgs like F-ado [30].

Two hydrogen bonds (N^6^-Asp^203^O^δ^ and N^7^-Ser^202^O^γ^) observed in BsPNP233-F-Ado complex, but not in BsPNP233-Ado complex, contribute for subtle changes in nucleoside position and base orientation (Figure 4E). The F-Ado ribosyl moiety adopts the catalytically favorable C4′-*endo* conformation supported by tight hydrogen bonds of nucleoside sugar hydroxyl groups with His^4*^, Arg^87^ and Glu^180^ side chains (Figure 4E). Structural comparisons among BsPNP233-F-Ado, TvPNP-F-Ado (PDB code 1Z35) and EcPNP-F-Ado (PDB code 1PK9) complexes showed a similar binding mode. However, the presence of phosphate in the EcPNP-(F-Ado) complex displaces by about 0.5 Å the ribosyl moiety, disrupts the N^7^-Ser^202^O^γ^ hydrogen bond and leads the N^7^ atom closer to the Asp^203^ side chain, favoring the catalysis (Figure 4F).

Since F-Ado binds to the BsPNP233 active site in a manner similar to that of the natural substrate Ado, placing the F^2^ atom in a hydrophobic pocket conserved in EcPNP, our structural data indicate that, as well as EcPNP [54], BsPNP233 is able to convert 2-fluoradenosine in the cytotoxic 2-fluoradenine. Thus, we concluded that BsPNP233 can be explored as an alternative in the development of anti-tumors gene therapy approaches using this prodrug or the less toxic 2-fluoro-2′-deoxyadenosine [57].

### The Cl^6^ Substituent of 6-chloroguanosine Induces a Ribose C3′-exo Conformation and May Prevent Catalysis

The NA 6-chloroguanosine (Cl-Guo) can be used for the synthesis of 2-amino-6-chloro-9-(2,3-dideoxy-3-fluoro-beta-D-erythro-pentofuranosyl)purine, a compound with anti-HBV effects [58]. In addition, the free base 6-chloroguanine is an inhibitor of the trimeric PNP from *Schistosoma mansoni* [59]. Here we report the first crystal structure of a PNP in complex with Cl-Guo.

**Figure 5:**
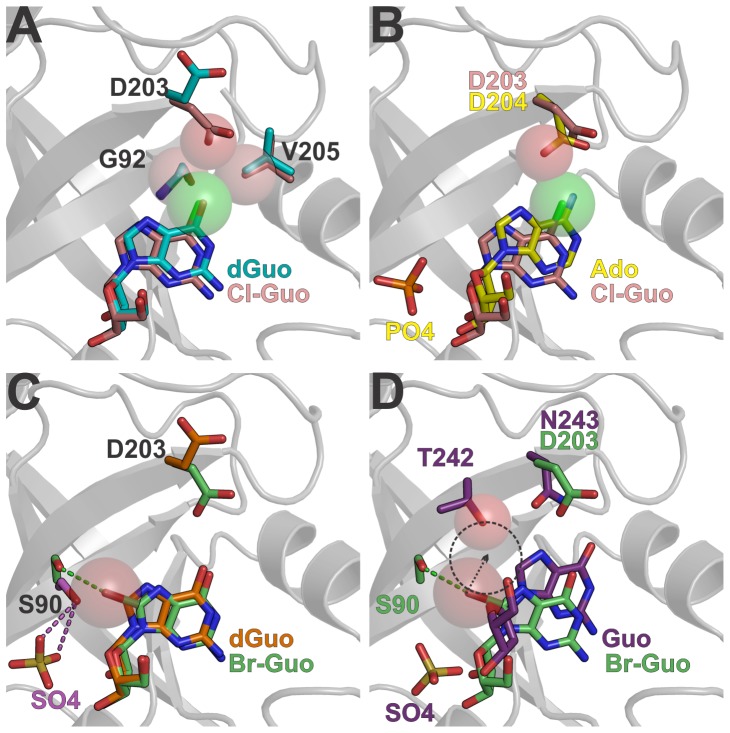
The influence of Cl^6^ and Br^8^ modifications in catalysis and nucleoside binding. A. Structural alignment of Cl-Guo complex (*pink* carbon atoms) and dGuo complex (*cyan* carbon atoms). Spheres represent the van der Waals radius of Cl^6^, Gly^92^C^α^, Asp^203^O^δ1^ and Val^205^C^γ2^ atoms. B. Superposition of Cl-Guo complex and EcPNP-Ado-PO_4_ complex (*yellow* carbon atoms, PDB code 1PK7 [54]). Spheres represent the van der Waals radius of Cl^6^ and EcPNP Asp^204^O^δ1^ atoms to highlight the steric conflict imposed by the Cl^6^ atom. C. The Br-Guo complex (carbon atoms in *green*), dGuo complex (carbon atoms in *orange*) and sulfate complex (carbon atoms in *magenta*, form IV, chain B) structures are superimposed. The sphere represents the van der Waals radius of Br^8^ and the dashed lines represent hydrogen bonds colored according to the respective structures. D. Structural comparison of Br-Guo complex and the trimeric HsPNP-Guo-SO_4_ complex (*purple* carbon atoms, PDB code 1RFG, [63]). The spheres represent the van der Waals radius of Br^8^ and HsPNP Thr^242^O^γ1^ atoms. The dashed circle has the same radius of Br^8^ and indicates the steric clash that would occur if BrGuo was placed at the Guo position in the HsPNP active site.

The molecule Cl-Guo displays a similar binding mode to that observed for dGuo (Figure 5A). However, as the chlorine van der Waals radius is larger than that of oxygen, the Cl^6^ substituent pushes the base in the direction of the ribosyl moiety to avoid steric clashes with Gly^92^C^α^, Val^205^C^γ2^, and Asp^203^O^δ1^ atoms. This base displacement induces the ribosyl group to adopt an unusual C3′-*exo* conformation.

The C3′-*exo* pucker was already observed in the nucleoside 9-β-D-xylofuranosyladenine bound to EcPNP (PDB code 1PR6, [54]) and it is considered incompatible with the sugar conformation required for PNP catalysis [54]. Moreover, structural comparisons with the EcPNP-Ado-PO_4_ complex (PDB code 1PK7, [54]) showed that the chlorine atom may prevent the Asp^203^ side chain to approach to the N^7^ atom to donate a proton during catalysis (Figure 5B). Thus, these findings suggest that Cl-Guo as well as other NAs with 6-substituents heavier than chlorine cannot be cleaved by BsPNP233 and other hexameric PNPs.

### The Br^8^ Substituent Displaces Ser^90^ Away from the Phosphate Binding Site and Might be Detrimental for Catalysis

The 8-bromoguanosine (Br-Guo) is a “poor substrate” of the trimeric PNP from calf spleen [60]. Its first crystallographic portrayal in complex with a protein is described here. The addition of a bromine radical at the C^8^ atom of guanosine results in the formation of a halogen bond between Br^8^ and Ser^90^O^γ^ atoms, which implicates in both positional and rotational displacement of the base by 0.3 Å and 7°, respectively (Figure 5C). The ribosyl moiety of Br-Guo presents a typical C4′–*endo* conformation and binds to the active site in a very similar fashion to that seen for 2′deoxyguanosine (Figure 5C).

In BsPNP233-(Br-Guo) complex, the side chain of the catalytic residue Asp^203^ is facing the O^6^ and N^7^ atoms of Br-Guo and the Ser^90^ side chain is pushed away from the active site in order to accommodate the bromine atom (Figure 5C). In hexameric PNPs, the hydroxyl group of Ser^90^ participates in the coordination of phosphate [61]. The position that it assumes in BsPNP233-sulfate and EcPNP-Ado-PO_4_ complexes (PDB code 1PK7 [54]) is incompatible with the presence of Br^8^ atom because of steric hindrance (Figure 5C). Thus, the bromine radical probably prevents the phosphate-Ser^90^ interaction being detrimental for binding and correct orientation of phosphate.

Site-directed mutations of human PNP phosphate binding site leads to a decrease in catalytic efficiency ranging from 25- to 185-fold [62]. Likewise, impairment of any phosphate interaction in the hexameric PNP active site may reduce the catalytic activity. As Br-Guo probably prevents the phosphate-Ser^90^ interaction it might be a “poor substrate” or even an inhibitor of BsPNP233 and other hexameric PNPs as well.

This interpretation cannot be applied for trimeric PNPs because Ser^90^ is not structurally conserved in trimeric PNPs. However, structural comparisons between BsPNP233-(Br-Guo) and human PNP in complex with guanosine and sulfate (PDB code 1RFG, [63]) indicate that Br-Guo is probably a “poor substrate” for trimeric PNPs because of steric hindrance involving the bromine and the side chain of Thr^242^, which would hinder the displacement of the N^7^ atom towards Asn^243^ side chain for stabilization of the transition state [64] (Figure 5D).

### Corroboration of the Competitive Inhibition Mechanism of Hexameric PNP by Tubercidin

Tubercidin (7-deazaadenosine) is an adenosine analogue which presents antiviral, antischistosomal and antifungal properties as well as antitumor activity [65]–[68]. Furthermore, tubercidin and other 7-deazapurine nucleosides are inhibitors of EcPNP [69], [70].

**Figure 6:**
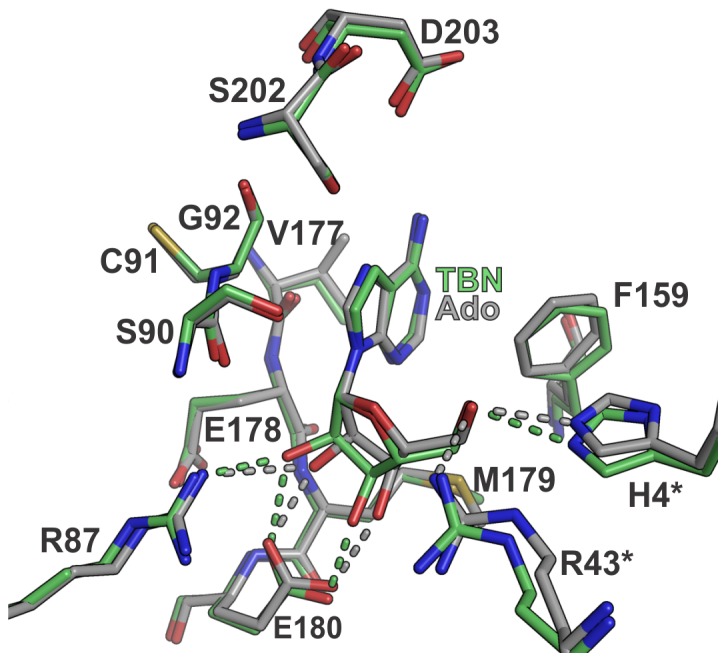
The binding mode of tubercidin. Structural comparison between the BsPNP233-TBN (carbon atoms in *green*) and BsPNP233-Ado (carbon atoms in *grey*) complexes. Dashed lines indicate hydrogen bonds and are colored according to their respective complexes.

TBN presented an interaction mode very similar to that seen for the natural substrate adenosine (Figure 6). Slightly differences were observed in its ribosyl moiety that assumed an O4′–*exo* pucker instead of the C3′-*exo* conformation of Ado in complex with BsPNP233 (Figure 6). The C^7^ substituent in TBN makes hydrophobic and van der Waals interactions with residues Cys^91^ and Ser^202^. The ribosyl moiety is stabilized by a conserved network of hydrogen bonds involving His^4*^, Glu^180^ and Arg^87^ side chains and by hydrophobic interactions with Glu^178^ (C^α^ and C^β^ atoms) and Met^179^C^γ^ atom (Figure 6). Our structural data corroborate the competitive inhibition mechanism of hexameric PNP by TBN defined by *in vitro* studies [69]. The substitution of N^7^ by a carbon prevents the protonation step of the N^7^ atom required for catalysis [70], making TBN a non-cleavable adenosine analogue by EcPNP and probably by other PNPs.

### Ganciclovir Inhibits Both Trimeric and Hexameric PNPs

**Figure 7:**
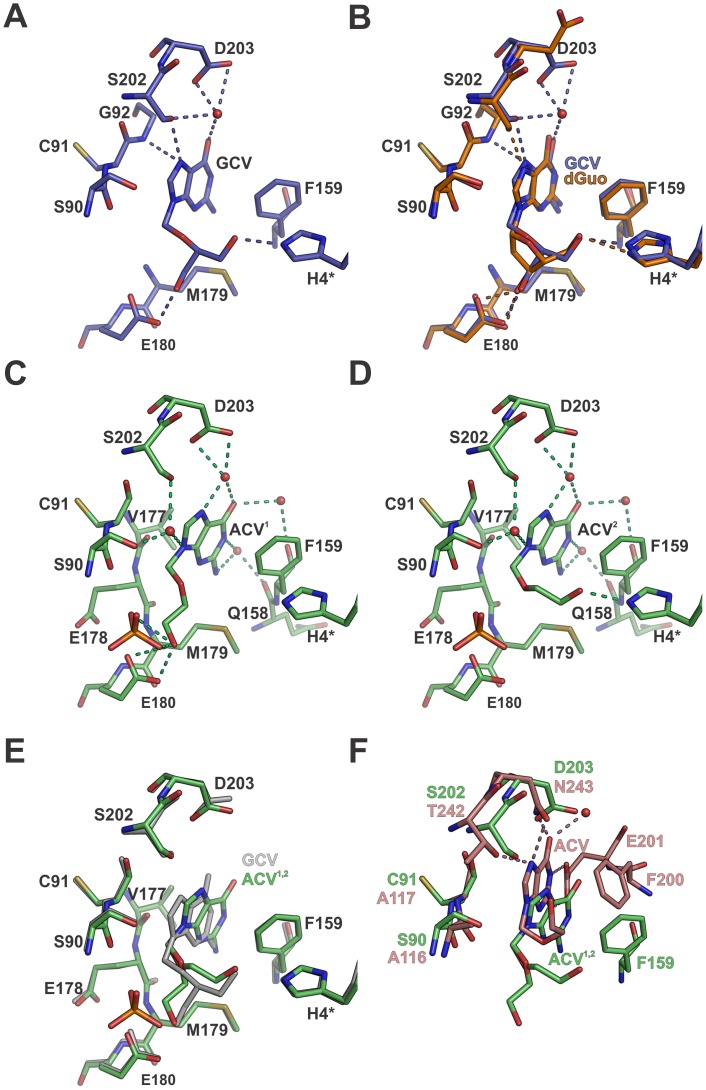
The binding mode of acyclic nucleosides. A. Stick representation of GCV bound in the BsPNP233 active site. B. Structural comparison of GCV-complex (*blue* carbon atoms) with dGuo-complex (*orange* carbon atoms). C and D show the stick representation of the two conformations of ACV (ACV^1^ and ACV^2^) bound to the BsPNP233 active site. E. The structures of GCV-complex (*grey*) and ACV^1,2^-complex (*green* carbon atoms) are superimposed. F. Structural alignment of ACV^1,2^-complex with HsPNP-ACV complex (*pink* carbon atoms, PDB code 1PWY [74]). In all panels dashed lines indicate hydrogen bonds and are color coded according to their respective complexes.

Ganciclovir (GCV) is an acyclic NA used to treat cytomegalovirus infections [17]. It is also used together with herpes simplex virus thymidine kinase in a suicide gene therapy system that has been studied for the treatment of hepatocellular carcinoma [71]. GCV is an inhibitor of the human PNP (trimeric) [72] and probably has inhibitory effects on hexameric PNPs as well. Our structural data support this hypothesis revealing that GCV binds to the nucleoside binding site of BsPNP233 (Figure 7).

The guanine moiety of GCV conserves the position observed for the 2′-deoxyguanosine base but it is rotated by about 10° to accommodate the acyclic chain in the ribose-binding site (Figure 7A–B). A water molecule mediates hydrogen bonds between the ligand O^6^ atom and the side chains of Ser^202^ and Asp^203^. The N^7^ atom interacts with Ser^202^O^γ^ and Gly^92^N atoms, and the base is stabilized by hydrophobic contacts with Ser^90^, Cys^91^, Ser^202^ and Phe^159^ (Figure 7A).

Interestingly, the three oxygens of the acyclic radical occupy similar positions to those observed for the three oxygens of dGuo ribosyl group, mimetizing its binding mode (Figure 7B). From the three hydrogen bonds observed for dGuo ribosyl moiety, the GCV acyclic radical conserves two, involving the His^4*^ and Glu^180^ side chains. Moreover, the C^4′^ atom of the GCV acyclic moiety preserves the hydrophobic interactions with Met^179^C^β^ and Met^179^C^γ^ atoms performed by the dGuo C^3′^ atom (Figure 7B). Therefore our data indicate that GCV is also a competitive inhibitor for hexameric PNPs.

### Aciclovir Acyclic Chain Adopts Two Conformations in the BsPNP233 Ribosyl Binding Site

Aciclovir (ACV) is an antiviral drug used to treat herpes virus infections [73] and has modest inhibitory effects on human PNP [74]. Here, we present for the first time the crystal structure of a hexameric PNP with ACV. This structure revealed differences in the aciclovir binding mode, which can be explored for drug design targeting hexameric PNPs from pathogens such as *P. falciparum* [6] and *T. vaginalis* [8].

Aciclovir binds to the BsPNP233 nucleoside binding site and is stabilized by hydrophobic interactions and a hydrogen-bonding network mediated by solvent molecules (Figure 7C–D). Interestingly, the acyclic tail assumes two alternative conformations that, seen simultaneously, resemble the conformation observed for the ganciclovir acyclic radical (Figure 7C–E). In one of these conformations, the 3′ hydroxyl group of ACV is attached to the carboxyl group of Glu^180^ side chain while the carbon atoms make hydrophobic contacts with the main chain of Glu^178^ and with the Met^179^C^β^ and Met^179^C^γ^ atoms (Figure 7C). A phosphate ion, modeled with half occupancy based on difference maps, also makes a hydrogen bond with the ligand 3′ hydroxyl group (Figure 7C). The other conformation is stabilized by a hydrogen bond between the 3′-OH group of ACV and the His^4*^ side chain (Figure 7D).

The ACV guanine moiety assumes a different position and orientation from that observed for GCV (Figure 7E), getting closer to the Phe^159^ side chain. The main chain of Cys^91^ and the side chain of Val^177^ also contribute with hydrophobic interactions to the base (Figure 7C–D). The O^6^ atom makes water mediated hydrogen bonds with the Asp^203^ side chain and with the Phe^159^ carbonyl oxygen (Figure 7C and D). The same is observed for N^1^ and N^2^ atoms, which interact through a water molecule with the Gln^158^ carbonyl oxygen; for N^7^ atom, which makes water mediated hydrogen bonds with Asp^203^ side chain, and; for N^9^ atom, whose interaction with both Ser^90^ and Ser^202^ hydroxyl groups is also mediated by a solvent molecule (Figure 7C–D).

Structural comparison between BsPNP233-ACV and human PNP (HsPNP)-ACV (PDB code 1PWY, [74]) complexes showed differences in the binding mode. In the HsPNP-ACV complex, the base N^1^, N^2^, N^7^ and O^6^ atoms interact directly with active-site residues through hydrogen bonds. In addition, the acyclic chain adopts a different conformation, which is stabilized by hydrophobic interactions with Phe^200^ side chain and Ala^116^/Ala^117^ main chains (Figure 7F). To investigate if differences in the interaction mode of aciclovir with BsPNP233 and HsPNP may result in different binding affinities, we estimated the strength of protein–ligand interactions using the rerank score function of MOLEGRO [75]. According to this analysis, ACV presented similar predicted binding affinities in both complexes, which was slightly higher (lower rerank score value) for the BsPNP233 complex (Table S3). The same analysis was performed for GCV whose predicted binding affinity was considerable higher than that observed for ACV (Table S3). This result indicates that GCV is a better inhibitor for hexameric PNPs than ACV.

### Structural Basis of Distinct Kinetic Models for Phosphate Binding in Hexameric PNPs

**Figure 8:**
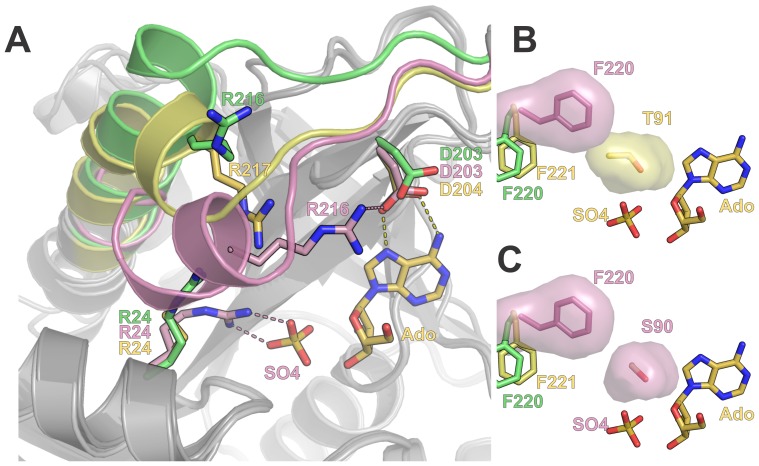
Structural basis of distinct kinetic models for phosphate binding in hexameric PNPs. A. Structural superposition of BsPNP233-sulfate open (*green*) and closed (*pink*) conformations with the BcAdoP-Ado complex (*yellow*, PDB code 3UAW, [52]). The cartoon representation highlights the conformational differences observed in the main chain of the β9-α7 loop and the N-terminal portion of helix α7 in the three structures. Dashed lines represent hydrogen bonds and follow the color code of their respective structures. B. The surface representation of BsPNP233 Phe^220^ in the closed conformation (*pink*) and of the BcAdoP Thr^91^ evidence the steric hindrance imposed by the Thr^91^C^γ2^ atom to that Phe^220^ rotamer. C. The surface representation of BsPNP233 Phe^220^ and Ser^90^ in the closed conformation shows that the Ser^90^ side chain allows the Phe^220^ side chain to perform the conformational change needed for the closed conformation takes place.

The asymmetric unit of the BsPNP233 crystal structure belonging to the *H*32 space group presented a catalytic dimer whose protomers adopt an open and a closed conformation, respectively (Figure 8A). The electron density map clearly showed a tetrahedral molecule in the active site of both subunits (Figure S1). As the crystallization condition was phosphate free and contained high concentrations of ammonium sulfate, we modeled sulfate ions in both sites.

The open and closed conformations of BsPNP233-sulfate complex were already observed in EcPNP-sulfate/phosphate structures and have been associated with two dissociation constants that characterize phosphate binding to EcPNP [61], [76]. The closed conformation is defined by a disruption of helix α7 and subsequent displacement of its N-terminal portion and the precedent loop towards the active site (Figure 8A). This conformation seems to be triggered by the interaction of Arg^24^ side chain with phosphate and results in an approximation of Arg^216^ to the catalytic residue Asp^203^ (Figure 8A) [61]. As BsPNP233 protomers are able to adopt open and closed conformations like EcPNP subunits, this suggests that the negative cooperativity of phosphate binding demonstrated for EcPNP [61] is also applied for BsPNP233.

Comparison between BsPNP233-sulfate and *Bacillus cereus* adenosine phosphorylase (BcAdoP)-sulfate complexes (PDB codes 3UAV, 3UAW, 3UAX, 3UAY, 3UAZ, [52]) showed that BcAdoP assumes an intermediate conformation where only the first turn of helix α7 is disrupted (Figure 8A). In the BcAdoP-sulfate complex structure (PDB code 3UAW, [52]), Arg^217^ (corresponding to BsPNP233-Arg^216^) points to the active site but it is not able to approach Asp^204^ (BsPNP233-Asp^203^) such as BsPNP233-Arg^216^ (Figure 8A).

The apparent inability of BcAdoP to adopt the closed conformation seems to be caused by a steric hindrance imposed by Thr^91^ to the conformational change that Phe^221^ (BsPNP233-Phe^220^) undergoes for the closed conformation being achieved (Figure 8B). In BsPNP233 and EcPNP this threonine residue is replaced by a serine, which allows Phe^220^ side chain to adopt the rotamer observed in the closed conformation (Figure 8C). These analyses suggest that the negative cooperativity model of phosphate binding displayed by EcPNP cannot be applied for BcAdoP, as BcAdoP apparently presents only one conformational state. This hypothesis is supported by functional studies, which showed that BcAdoP obeys Michaelis–Menten kinetics [77].

A previous work reported that BsPNP233 is specific for 6-aminopurine nucleosides [78]. However, Xie and coworkers [34] recently showed that BsPNP233 (named PNP_702_) exhibits a broad substrate specificity and present comparable activity towards both guanosine (6-oxopurine nucleoside) and adenosine (6-aminopurine nucleoside). Our structural data is in agreement with Xie and coworkers data indicating that BsPNP233 conserves the same catalytic mechanism proposed for EcPNP [76], where catalysis occurs in the closed conformation (Figure 8A).

### Conclusion

This report provided a broad description of how the hexameric PNP from *B. subtilis* interacts with natural substrates and the impact of modifications in such substrates on binding and catalysis. The structural analysis reported here can be instrumental for studies aiming to optimize BsPNP233 or other hexameric PNPs for biotechnological applications such as industrial synthesis of nucleoside analogues or gene therapy against solid tumors. An initiative of this sort has been taken for *E. coli* PNP to optimize the cleavage of the prodrug Me(*talo*)-MeP-R with great success [29].

The crystal structure of six ligands (adenine, 2′deoxyguanosine, aciclovir, ganciclovir, 8-bromoguanosine and 6-chloroguanosine) in complex with a hexameric PNP are presented for the first time. The information extracted from these structures can be extended to homologous hexameric PNPs to help the development of new inhibitors against pathogens such as *T. vaginalis* [8] and *P. falciparum* [6] as well as new prodrugs for gene therapies against tumors [30], [79].

In addition, our results and comparative analyses shed light on distinct kinetic models for phosphate binding in hexameric PNPs. According to our model the substitution of the conserved residue Ser^90^ by a threonine disrupts the open/close mechanism of hexameric PNPs subunits, which results in the loss of the negative cooperativity of phosphate binding.

## Supporting Information

Figure S1 **Weighted 2Fo-Fc map (2mFo-DFcalc) of the ligands (** ***ball and stick*** **) bound to the BsPNP233 active site.** A. Ade-complex (chain A). B. Ade-SO_4_ complex, evidencing only Ade (form I, chain A). C. Hyp-complex. D. Ado-complex (chain A). E. F-Ado complex (chain A). F. dGuo complex. G. Cl-Guo complex. H. Br-Guo complex. I. TBN complex. J. GCV complex. K. ACV complex. L. SO_4_ complex (form IV, chain A).
(TIF)

Figure S2 **Crystallographic interfaces (** ***dark grey*** **) observed at the crystal structures solved at space groups** ***P*** **32_1_,** ***P*** **2_1_2_1_2_1_,** ***P*** **6_3_22 (A) and at** ***H*** **32 space group (B).**
(TIF)

Figure S3 **Structural alignment of BsPNP233 subunit with homologous hexameric PNPs protomers.** The regions with the highest r.m.s.d. values are colored: BsPNP233 (*green*), BaPNP (*blue* - PDB 1XE3/F), BcPNP (*yellow* - PDB 2AC7/B), EcPNP (*red* - PDB 1ECP/A).
(TIF)

Table S1 **Data collection and refinement statistics.**
(DOC)

Table S2 **Distances (Å) between the ligand atoms and interacting BsPNP233 atoms.** Potential hydrogen bonds are highlighted by grey boxes. In the case of crystal structures containing more than one complex per asymmetric unit, only one of them is shown in the table. For the ligand adenine a representative structure of the preferential^§^ (BsPNP233-Ade complex, chain A) and alternative^¥^ (form I, chain A) conformations are presented.
(DOC)

Table S3 ***In silico*** **prediction of ligand binding affinity using the rerank score function of MOLEGRO** [**75**] **.** ^¥^ The two values of BsPNP233-ACV complex correspond to the ACV^1^ and ACV^2^ alternative conformations, respectively.^ §^ HsPNP-ACV (PDB CODE: 1PWY).
(DOC)
