# Supplementary figures and images for: Insights into Phosphate Cooperativity and Influence of Substrate Modifications on Binding and Catalysis of Hexameric Purine Nucleoside Phosphorylases
Source: PLoS One. 2012 Sep 5;7(9):e44282. doi: 10.1371/journal.pone.0044282 (PMC3434127; doi:10.1371/journal.pone.0044282)

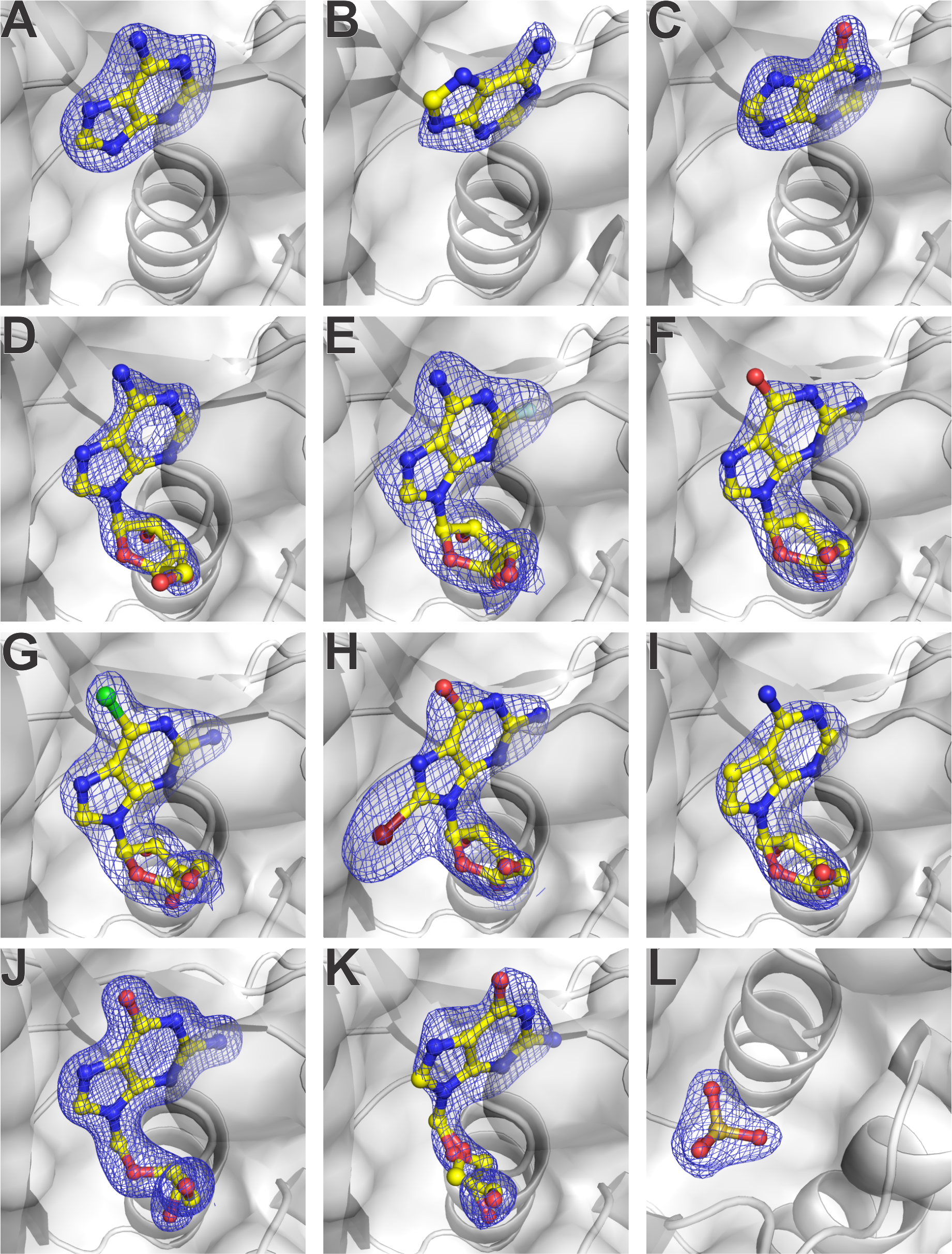

Supplement: Figure S1 — Weighted 2Fo-Fc map (2mFo-DFcalc) of the ligands ( ball and stick ) bound to the BsPNP233 active site. A. Ade-complex (chain A). B. Ade-SO4 complex, evidencing only Ade (form I, chain A). C. Hyp-complex. D. Ado-complex (chain A). E. F-Ado complex (chain A). F. dGuo complex. G. Cl-Guo complex. H. Br-Guo complex. I. TBN complex. J. GCV complex. K. ACV complex. L. SO4 complex (form IV, chain A). (TIF) [file pone.0044282.s001.tif]

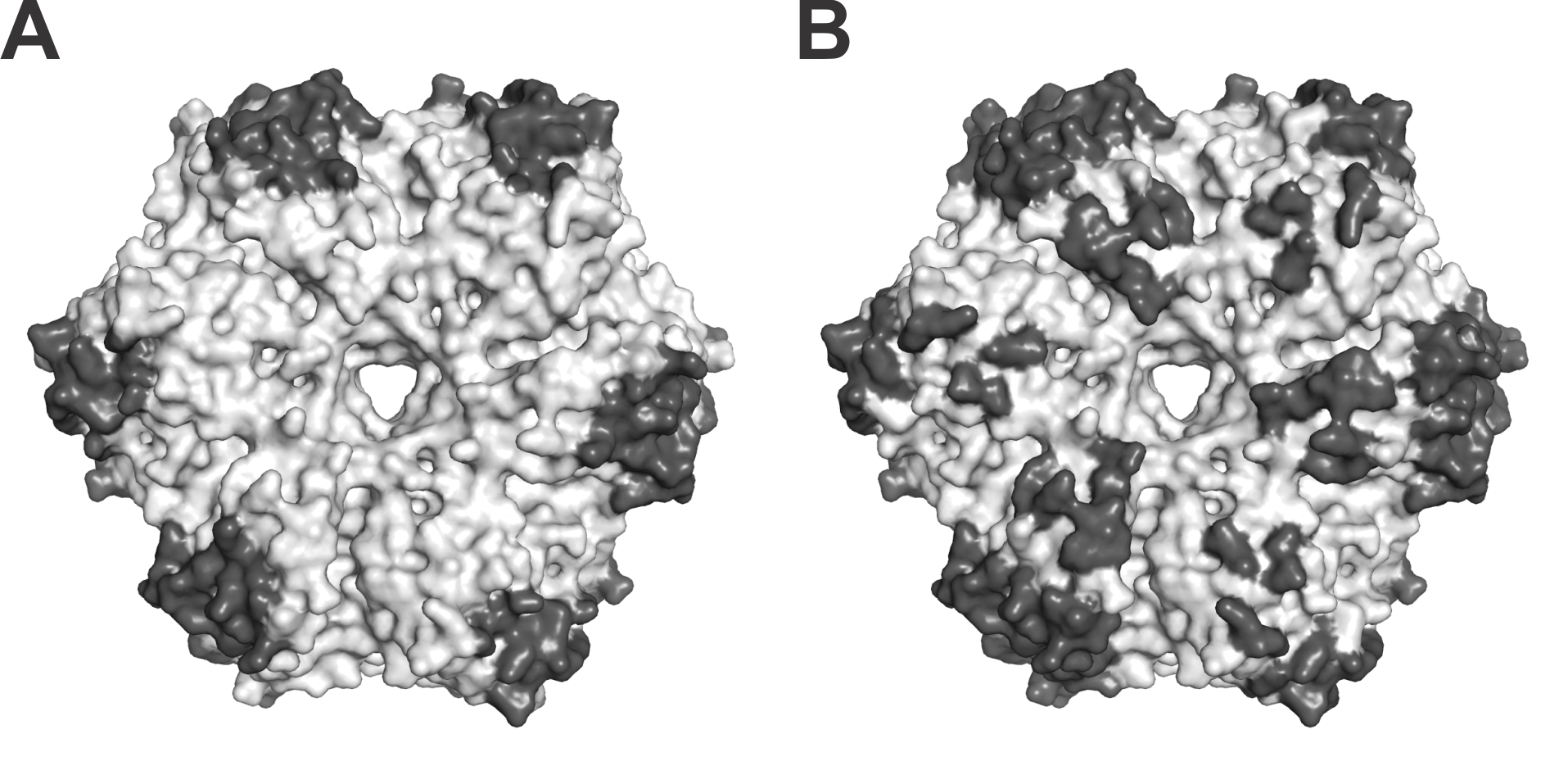

Supplement: Figure S2 — Crystallographic interfaces ( dark grey ) observed at the crystal structures solved at space groups P 321, P 212121, P 6322 (A) and at H 32 space group (B). (TIF) [file pone.0044282.s002.tif]

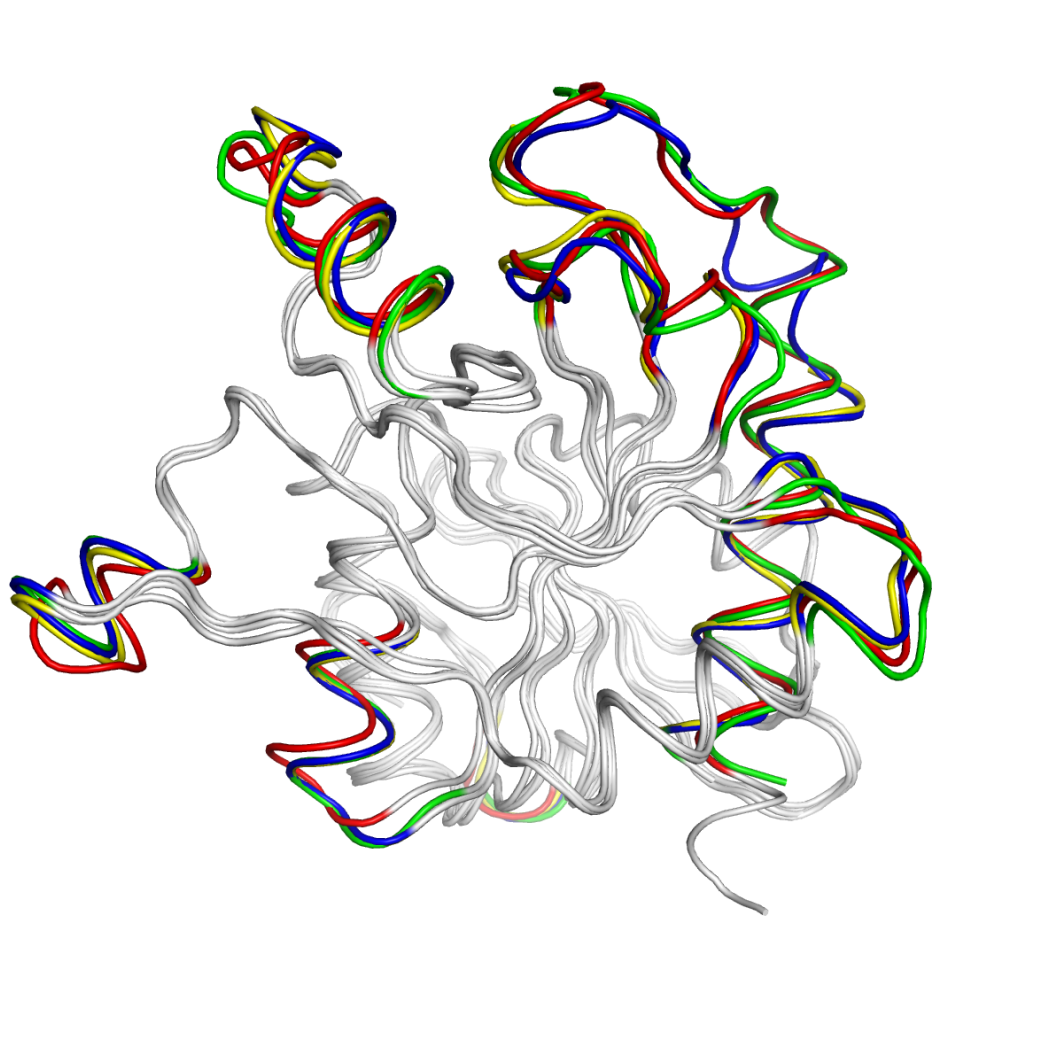

Supplement: Figure S3 — Structural alignment of BsPNP233 subunit with homologous hexameric PNPs protomers. The regions with the highest r.m.s.d. values are colored: BsPNP233 (green), BaPNP (blue - PDB 1XE3/F), BcPNP (yellow - PDB 2AC7/B), EcPNP (red - PDB 1ECP/A). (TIF) [file pone.0044282.s003.tif]
