# Supplementary material for: Insights into Phosphate Cooperativity and Influence of Substrate Modifications on Binding and Catalysis of Hexameric Purine Nucleoside Phosphorylases
Source: PLoS One. 2012 Sep 5;7(9):e44282. doi: 10.1371/journal.pone.0044282 (PMC3434127; doi:10.1371/journal.pone.0044282)
Supplement: Table S1 — Data collection and refinement statistics. (DOC) [file pone.0044282.s004.doc]

|  | I | II | III | IV | BsPNP233 – Hyp | BsPNP233 – Ade | BsPNP233 – Ado |
| --- | --- | --- | --- | --- | --- | --- | --- |
| **PDB code**  **Data collection** | 4D8V | 4D8X | 4D8Y | 4D98 | 4DAB | 4DAO | 4D9H |
| Space group | *P*32_1_ | *P*6_3_22 | *P*2_1_2_1_2_1_ | *H*32 | *P*6_3_22 | *P*32_1_ | *P*32_1_ |
| Cell dimensions   |  |  |  |  |  |  |  |
| *a*, *b*, *c* (Å) | 136.83, 136.83, 57.11 | 135.81, 135.81, 57.02 | 56.79, 135.98, 236.61 | 158.26, 158.26, 93.87 | 135.39, 135.39, 58.01 | 136.46, 136.46, 55.48 | 135.72, 135.72, 56.10 |
|     α, β, γ  (°) | 90, 90, 120 | 90, 90, 120 | 90, 90, 90 | 90, 90, 120 | 90, 90, 120 | 90, 90, 120 | 90, 90, 120 |
| Molecules per AU^§^ | 2 | 1 | 6 | 2 | 1 | 2 | 2 |
| Resolution (Å) | 40-2.35 (2.43-2.35) | 30-2.65 (2.74-2.65) | 50-1.61 (1.67-1.61) | 30-1.70 (1.76-1.70) | 50-1.85 (1.92-1.85) | 40-2.22 (2.30-2.22) | 40-1.91 (2.01-1.91) |
| *R*_merge_ | 9.2 (48.8) | 10.0 (48.0) | 4.8 (18.7) | 9.3 (38.2) | 5.7 (51.1) | 4.4 (28.5) | 8.2 (55.9) |
| *<I* / σ*I>* | 14.0 (3.8) | 18.0 (3.8) | 28.5 (4.7) | 22.4 (4.6) | 28.2 (3.8) | 33.3 (5.0) | 14.6 (3.1) |
| Completeness (%) | 98.3 (97.4) | 99.1 (99.9) | 98.8 (93.4) | 95.5 (92.6) | 97.2 (91.0) | 99.8 (98.3) | 100.0 (100.0) |
| Multiplicity | 7.6 (7.6) | 6.4 (6.2) | 3.3 (2.5) | 8.0 (7.0) | 6.9 (6.4) | 5.6 (5.2) | 6.3 (6.2) |
|  |  |  |  |  |  |  |  |
| **Refinement** |  |  |  |  |  |  |  |
| Resolution (Å) | 20.00-2.35 | 29.17-2.65 | 20.0-1.61 | 29.81-1.70 | 41.23 – 1.85 | 20.0-2.22 | 20.0-1.91 |
| No. reflections | 23731 | 8841 | 222700 | 44576 | 24968 | 27961 | 43947 |
| *R*_work_ / *R*_free_ | 0.225/0.275 | 0.192/0.262 | 0.166/0.190 | 0.168/0.206 | 0.201 / 0.241 | 0.148/0.208 | 0.145/0.175 |
| No. atoms | 3665 | 1821 | 12418 | 3954 | 2003 | 3593 | 3674 |
| Protein | 3525 | 1778 | 11316 | 3602 | 1840 | 3519 | 3558 |
| Ligand/ion | 40 | - | 138 | 18 | 33 | 20 | 19 |
| Water | 100 | 43 | 964 | 334 | 130 | 54 | 97 |
| Mean *B*-factors (Å^2^) | 49.44 | 40.02 | 22.81 | 24.03 | 29.73 | 40.36 | 26.52 |
| Protein | 49.38 | 40.27 | 21.94 | 23.12 | 29.11 | 40.28 | 26.45 |
| Ligand/ion | 55.11 | - | 27.16 | 34.30 | 44.50 | 46.11 | 30.59 |
| Water | 49.32 | 29.86 | 32.36 | 33.25 | 34.71 | 43.70 | 28.13 |
| R.m.s. deviations |  |  |  |  |  |  |  |
| Bond lengths (Å) | 0.013 | 0.015 | 0.015 | 0.016 | 0.016 | 0.015 | 0.014 |
| Bond angles (°) | 1.365 | 1.506 | 1.572 | 1.557 | 1.528 | 1.517 | 1.359 |
| Ramachandran Plot |  |  |  |  |  |  |  |
| Favored (%) | 97.2 | 97.8 | 99.2 | 99.1 | 99.6 | 98.7 | 98.5 |
| Allowed (%) | 2.8 | 2.2 | 0.8 | 0.9 | 0.4 | 1.1 | 1.5 |
| Disallowed (%) | 0.0 | 0.0 | 0.0 | 0.0 | 0.0 | 0.2 | 0.0 |

|  | BsPNP233 – dGuo | BsPNP233 – F-Ado | BsPNP233 – Cl-Guo | BsPNP233 – Br-Guo | BsPNP233 – TBN | BsPNP233 – GCV | BsPNP233 – ACV |
| --- | --- | --- | --- | --- | --- | --- | --- |
| **PDB code**  **Data collection** | 4DA0 | 4DAN | 4DAE | 4DA8 | 4DAR | 4DA6 | 4DA7 |
| Space group | *P*6_3_22 | *P*32_1_ | *P*6_3_22 | *P*6_3_22 | *P*6_3_22 | *P*6_3_22 | *P*6_3_22 |
| Cell dimensions   |  |  |  |  |  |  |  |
| *a*, *b*, *c* (Å) | 135.35, 135.35, 56.80 | 135.80, 135.80, 55.15 | 135.35, 135.35, 56.80 | 135.75, 135.75, 57.38 | 135.64, 135.64, 57.56 | 135.55, 135.55, 57.77 | 135.05, 135.05, 57.88 |
|     α, β, γ  (°) | 90, 90, 120 | 90, 90, 120 | 90, 90, 120 | 90, 90, 120 | 90, 90, 120 | 90, 90, 120 | 90, 90, 120 |
| Molecules per AU^§^ | 1 | 2 | 1 | 1 | 1 | 1 | 1 |
| Resolution (Å) | 50-2.95 (3.06-2.95) | 40-2.56 (2.70-2.56) | 50-2.35 (2.43-2.35) | 50-2.60 (2.69-2.60) | 40-3.15 (3.32-3.15) | 50-1.70 (1.76-1.70) | 50-2.05 (2.12-2.05) |
| *R*_merge_ | 12.7 (51.0) | 10.4 (30.3) | 10.9 (41.1) | 9.4 (53.7) | 11.6 (34.0) | 6.6 (45.5) | 8.1 (49.1) |
| *<I* / σ*I>* | 18.1 (4.0) | 12.3 (6.3) | 17.8 (4.2) | 20.8 (2.5) | 10.5 (5.5) | 29.3 (3.1) | 20.1 (3.2) |
| Completeness (%) | 99.7 (98.3) | 93.7 (93.7) | 99.9 (99.4) | 99.5 (96.1) | 99.8 (100.0) | 98.4 (98.3) | 99.5 (98.7) |
| Multiplicity | 10 (8.5) | 4.4 (4.4) | 9.9 (6.1) | 7.8 (4.9) | 5.3 (5.4) | 8.8 (4.8) | 5.7 (5.2) |
|  |  |  |  |  |  |  |  |
| **Refinement** |  |  |  |  |  |  |  |
| Resolution (Å) | 44.30 - 2.95 | 20.0-2.56 | 44.30 – 2.35 | 44.44-2.60 | 20.0-3.15 | 41.17 – 1.70 | 41.14 – 2.05 |
| No. reflections | 6461 | 16790 | 12524 | 9436 | 5453 | 32440 | 18808 |
| *R*_work_ / *R*_free_ | 0.227 / 0.309 | 0.157/0.211 | 0.195 / 0.248 | 0.203/0.260 | 0.217/0.271 | 0.178 / 0.204 | 0.192 / 0.232 |
| No. atoms | 1775 | 3549 | 1906 | 1827 | 1780 | 2040 | 1901 |
| Protein | 1755 | 3509 | 1802 | 1767 | 1761 | 1853 | 1783 |
| Ligand/ion | 20 | 40 | 37 | 21 | 19 | 33 | 26 |
| Water | 0 | 0 | 67 | 39 | 0 | 154 | 92 |
| Mean *B*-factors (Å^2^) | 62.90 | 23.59 | 37.35 | 49.11 | 60.80 | 28.78 | 32.55 |
| Protein | 62.72 | 23.55 | 37.22 | 49.40 | 60.85 | 28.08 | 32.43 |
| Ligand/ion | 78.75 | 26.93 | 45.83 | 47.12 | 56.61 | 34.76 | 38.68 |
| Water | - | - | 36.38 | 36.71 | - | 35.91 | 33.13 |
| R.m.s. deviations |  |  |  |  |  |  |  |
| Bond lengths (Å) | 0.011 | 0.013 | 0.015 | 0.015 | 0.009 | 0.014 | 0.017 |
| Bond angles (°) | 1.302 | 1.407 | 1.565 | 1.579 | 1.153 | 1.473 | 1.536 |
| Ramachandran Plot |  |  |  |  |  |  |  |
| Favored (%) | 93.0 | 97.8 | 98.3 | 96.1 | 95.6 | 99.1 | 99.1 |
| Allowed (%) | 7.0 | 2.2 | 1.7 | 3.9 | 4.4 | 0.9 | 0.9 |
| Disallowed (%) | 0.0 | 0.0 | 0.0 | 0.0 | 0.0 | 0.0 | 0.0 |

Values in parentheses are for highest resolution shell. ^§^AU, asymmetric unit
