# Supplementary material for: Insights into Phosphate Cooperativity and Influence of Substrate Modifications on Binding and Catalysis of Hexameric Purine Nucleoside Phosphorylases
Source: PLoS One. 2012 Sep 5;7(9):e44282. doi: 10.1371/journal.pone.0044282 (PMC3434127; doi:10.1371/journal.pone.0044282)
Supplement: Table S3 — In silico prediction of ligand binding affinity using the rerank score function of MOLEGRO [75] . ¥ The two values of BsPNP233-ACV complex correspond to the ACV1 and ACV2 alternative conformations, respectively. § HsPNP-ACV (PDB CODE: 1PWY). (DOC) [file pone.0044282.s006.doc]

| **Complex** | **Rerank score** |
| --- | --- |
| **BsPNP233-dGUO** | **-95.230** |
| **BsPNP233-GCV** | **-112.954** |
| **BsPNP233-ACV** | **-84.727/-87.752^¥^** |
| **HsPNP-ACV ^§^** | **-83.635** |


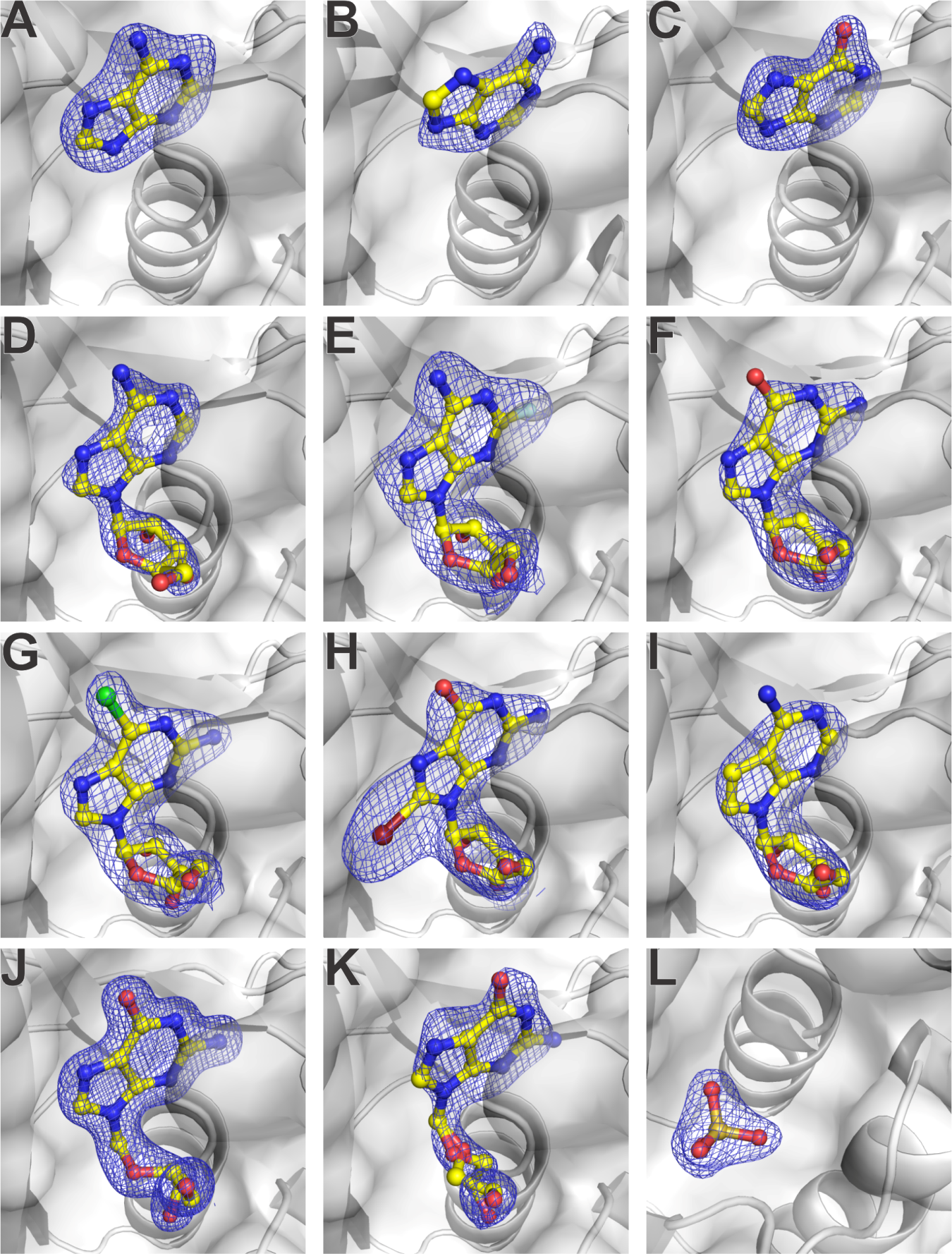


FIGURE S1. Weighted 2Fo-Fc map (2mFo-DFcalc) of the ligands (*ball and stick*) bound to the BsPNP233 active site. A. Ade-complex (chain A). B. Ade-SO_4_ complex (form I, chain A). C. Hyp-complex. D. Ado-complex (chain A). E. F-Ado complex (chain A). F.dGuo complex. G. Cl-Guo complex. H. Br-Guo complex. I. TBN complex. J. GCV complex. K. ACV complex. L. SO_4_ complex (form IV, chain A).


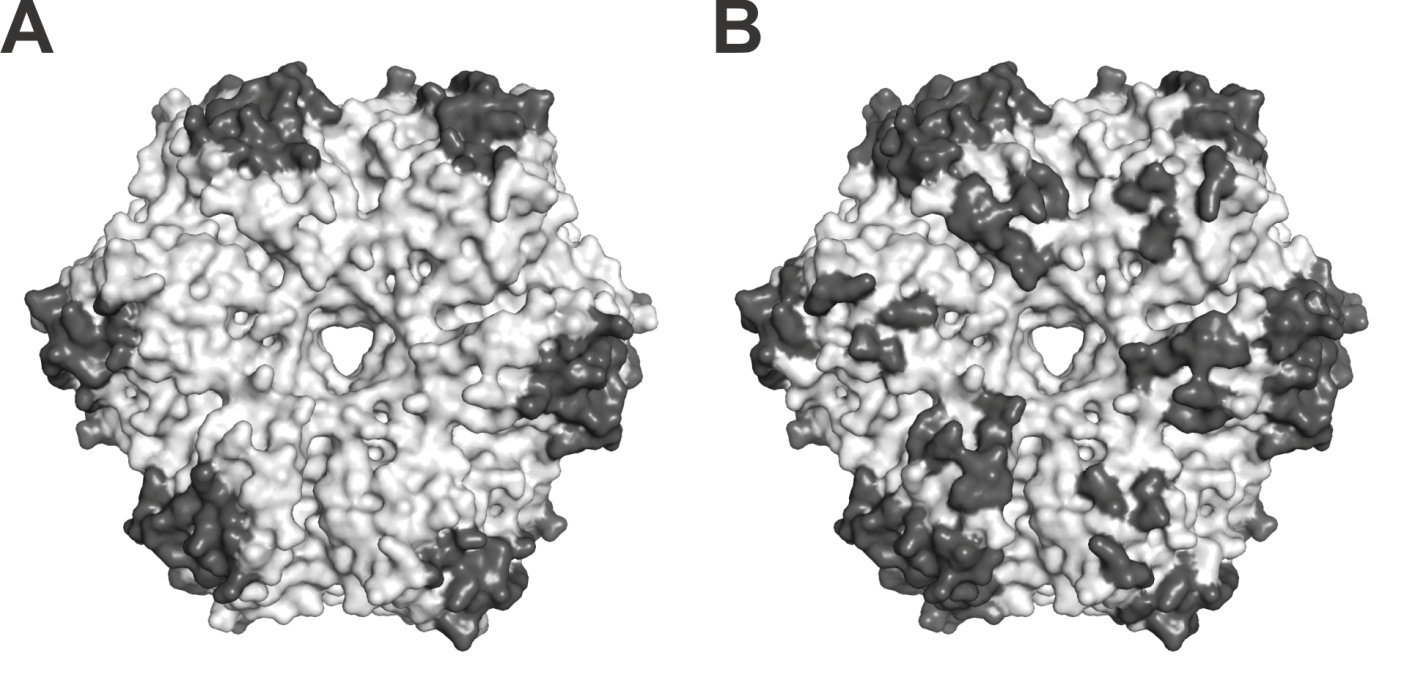


FIGURE S2. Crystallographic interfaces (*dark grey*) observed at the crystal structures solved at space groups *P*32_1_*, P*2_1_2_1_2_1_*, P*6_3_22 (A) and at *H*32 space group (B).


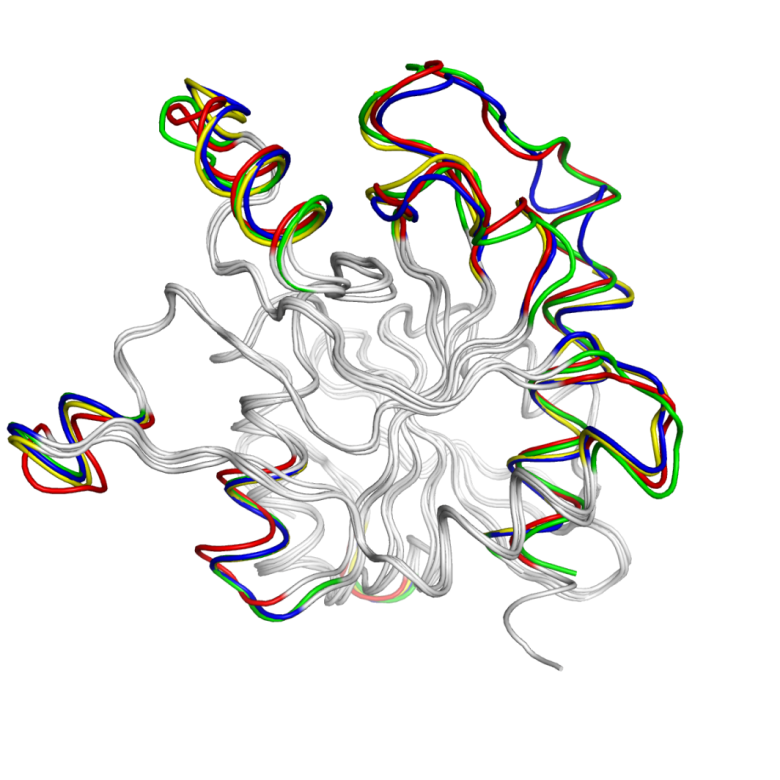


FIGURE S3. Structural alignment of BsPNP233 subunit with homologous hexameric PNPs protomers. The regions with the highest r.m.s.d. values are colored: BsPNP233 (*green*), BaPNP (*blue* - PDB 1XE3/F), BcPNP (*yellow* - PDB 2AC7/B), EcPNP (*red* - PDB 1ECP/A).
