# Supplementary material for: Insights into Phosphate Cooperativity and Influence of Substrate Modifications on Binding and Catalysis of Hexameric Purine Nucleoside Phosphorylases
Source: PLoS One. 2012 Sep 5;7(9):e44282. doi: 10.1371/journal.pone.0044282 (PMC3434127; doi:10.1371/journal.pone.0044282)
Supplement: Supplementary file 4 [file pone.0044282.s005.doc]

	lig. atom	BsPNP233	Hyp	Ade§	Ade¥	Ado	dGuo	F-Ado	Cl-Guo	Br-Guo	TBN	GCV	ACV[1]	ACV[2]
base	C2	PHE159/CD1	3.71	3.44	3.76	3.52	3.69	3.42	3.46	3.69	3.68	3.51	3.62	3.62
	C2	PHE159/CE1		3.68		3.75	3.79	3.45	3.65	3.79	3.77	3.77		
	C2	VAL177/CG2	3.63	3.81						3.85			3.57	3.57
	C2	GLU178/CA			3.70									
	C2	MET179/CG	3.41		3.43									
	C4	PHE159/CE1	3.73	3.60	3.62			3.80		3.73			3.73	3.73
	C5	PHE159/CD1	3.86											
	C5	PHE159/CE1	3.54	3.54	3.46			3.84		3.76			3.55	3.55
	C6	GLY92/CA					3.72	3.85						
	C6	PHE159/CD1	3.72		3.66									
	C6	PHE159/CE1	3.80	3.56	3.69			3.70	3.87	3.82	3.82	3.87	3.74	3.74
	C6	VAL177/CG1	3.86			3.89								
	N6	ASP203/OD1						3.13						
	C7	CYS91/C									3.71			
	C8	SER90/C				3.74	3.68	3.72	3.44			3.69		
	C8	CYS91/C	3.85	3.28		3.86						3.70	3.64	3.64
	C8	CYS91/CA		3.44		3.71	3.70	3.84	3.80		3.77	3.53		
	C8	GLY92/CA	3.73		3.80									
	C8	SER202/CB										3.86		
	N7	GLY92/N		3.19								3.22		
	N7	SER202/OG					2.68	2.63	2.80	3.11		2.75		
	Br8	SER90/OG								2.94				
ribosyl/acyclic radical	C1'	CYS91/CA											3.83	3.83
	C1'	GLU178/CA											3.74	3.76
	C2'	GLU178/CA					3.59			3.83	3.81			
	C2'	GLU178/CB					3.70				3.72			
	C2'	GLU178/C					3.82							
	C2'	MET179/CB								3.87				
	C2'	MET179/CG							3.86	3.71			3.66	
	O2'	ARG87/NH1				3.27		3.18	3.01	3.08	2.77			
	O2'	MET179/N				3.05		3.27	2.97	3.14				
	O2'	GLU180/OE1				2.54		2.66	2.40	2.58	2.81			
	O2'	GLU180/OE2						3.26						
	C3'	MET179/CB					3.76			3.74			3.83	
	C3'	MET179/CG					3.89			3.72	3.85			
	C3'	GLU180/CD											3.61	
	O3'	GLU180/OE1					2.64		3.03	2.81	2.82		3.24	
	O3'	GLU180/OE2				2.67	2.60	2.46	2.50	2.48	2.52		2.66	
	O3'	HIS4/NE2*										2.72		2.72
	C4'	MET179/CB										3.55		
	C4'	MET179/CG										3.51		
	C4'	GLU180/CD										3.82		
	O4'	GLU180/OE2										2.71		
	C5'	PHE159/CD1					3.64		3.72	3.87				
	C5'	PHE159/CE1				3.85	3.60	3.71	3.82	3.82				
	O5'	HIS4/NE2*				2.81	2.98	2.42	2.56	2.97	3.07			
	O5'	ARG43/NH2*				2.90		2.98						
